# Effect of Expansive Agent on Properties and Microstructure of Coal Gangue-Slag-Fly Ash Based Geopolymer

**DOI:** 10.3390/ma18194607

**Published:** 2025-10-04

**Authors:** Qi Wang, Mei Zhou, Xinyi Wang, Yang Han, Lei Peng, Gang Ma

**Affiliations:** 1 School of Civil Engineering, Liaoning Technical University, Fuxin 123000, China; 18031920426@189.cn (Q.W.); 14741444263@163.com (X.W.); hanyang0305@126.com (Y.H.); 2 Engineering Research Center of Coal-Based Solid Waste Utilization of Liaoning, Fuxin 123000, China; 3 Key Laboratory of Coal Gangue Resource Utilization and Energy-Saving Building Materials of Liaoning, Fuxin 123000, China; 4 School of Civil Engineering, Central South University, Changsha 410075, China; niclauspeng@163.com

**Keywords:** coal gangue-slag-fly ash based geopolymer, expansive agents, microstructure, shrinkage model

## Abstract

Expansive agents (CaO, MgO, C_4_A_3_Š) were incorporated into coal gangue-slag-fly ash based geopolymer (CSFG). The influence of expansive agents on the properties and microstructure of CSFG was investigated by macroscopic tests including setting time, compressive strength, and shrinkage values, along with microstructural tests including XRD, FTIR, SEM-EDS, and BET. Results showed that CaO and MgO added separately and their combination exhibited similar trends, with CaO added separately yielding the most favorable outcome. In comparison to the control group, the sample with 7% CaO reduced initial and final setting times by 43.6% and 52.8%, increased 28 d compressive strength by 12.6%, and decreased 28 d drying shrinkage and autogenous shrinkage values by 43.5% and 29.9%, respectively. Moderate MgO and CaO enhanced dissolution of precursors (e.g., coal gangue, fly ash), promoting formation of C-A-S-H gel, CaCO_3_, and periclase. Incorporating 3% C_4_A_3_Š shortened initial and final setting times by 41.3% and 17.8%, improved 28 d compressive strength by 32.2%, but increased 28 d drying and autogenous shrinkage values by 58.3% and 12.8%. Exceeding 3% content significantly reduced 3 d strength. Excessive C_4_A_3_Š promoted rapid ettringite (AFt) formation, leading to microcracking. Correction prediction models for drying shrinkage strain and autogenous shrinkage strain of CSFG were developed, demonstrating good agreement between predictive and actual values.

## 1. Introduction

The preparation of high-quality, low-carbon cementitious materials using industrial solid waste is of great significance for China’s “dual-carbon” national strategy. Geopolymers, due to their superior performance and eco-friendly attributes, are widely regarded as an important alternative to traditional cement. However, challenges such as uncontrollable setting time and greater drying shrinkage and autogenous shrinkage compared with cement-based materials have limited their development and large-scale application [1,2,3]. Early geopolymers were produced using calcined clay as the raw material, alkaline compounds as activators, and water as the mixing medium. Later studies found that solid wastes containing silica and alumina could replace calcined clay, and that combined activation by alkalis and salts produced better results, which gradually made geopolymer research a hotspot [4,5,6]. However, geopolymers still face key challenges in practical applications, including strong dependency on precursor materials, difficulties in controlling setting and shrinkage properties, susceptibility to alkali migration and efflorescence, and lack of standardized systems, which hinder their engineering promotion [7,8,9,10]. In recent years, numerous researchers have carried out modification studies, particularly focusing on setting time and early shrinkage, and found that incorporating alkaline earth metal activators yields notable effects. For example, Aydin and He et al. [11] demonstrated experimentally that calcium-rich materials effectively reduce the shrinkage rate of alkali-activated slag (AAS); Vandeperre et al. [12,13] further revealed that MgO expansive agents significantly reduce shrinkage in alkali-activated materials. It is noteworthy that alkaline earth metal activators such as C_4_A_3_Š, MgO, and CaO not only act as activators but also possess expansive properties; their hydration products compensate for geopolymer shrinkage, making them a promising choice of activator with more balanced overall performance.

In summary, this study employs a ternary blend of activated coal gangue powder, fly ash, and slag as the CSFG precursor [14,15], with a combined NaOH and Na_2_O·nSiO_2_ system as the alkaline activator. Expansive agents, including C_4_A_3_Š, MgO, CaO, and a MgO-CaO combination, are introduced individually and in binary mixes to investigate the effects of type and content on the performance and microstructure of CSFGs. To systematically evaluate the influence of expansive agents on the workability, mechanical properties, and volume stability of geopolymer, macroscopic property tests, including setting time determination, compressive strength measurement, and shrinkage assessment, were carried out. Meanwhile, microstructural characterization is carried out using XRD, FTIR, SEM-EDS, and BET techniques to elucidate the polymerization mechanisms of C(N)-A-S-H gels, AFt crystals, and related phases. Additionally, MATLAB is employed to perform multivariate nonlinear regression fitting of shrinkage parameters based on the GL-2000 and EN-1992 models, thereby establishing predictive models for early-age drying shrinkage and autogenous shrinkage. The feasibility and accuracy of these models are further validated, providing theoretical support for the practical application of multi-source solid waste—based geopolymers.

## 2. Experiment

### 2.1. Raw Materials and Sample Preparation

#### 2.1.1. Raw Materials

**Figure 1:**
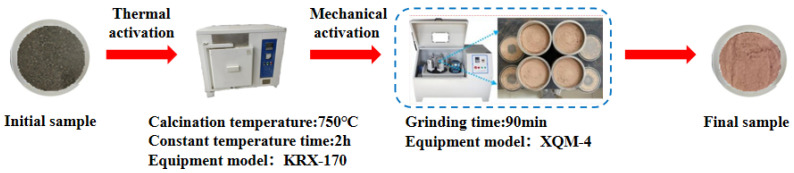
ACG preparation process.

**Figure 2:**
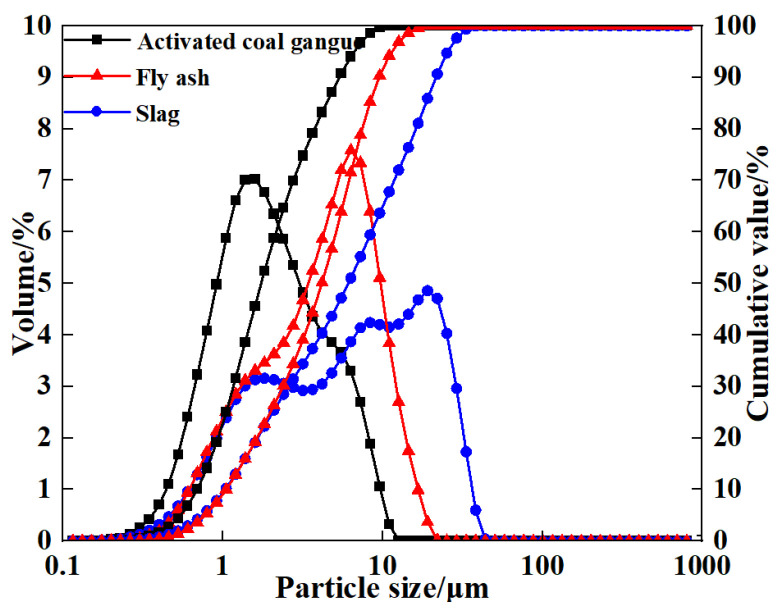
Particle size distribution of the precursor.

**Figure 3:**
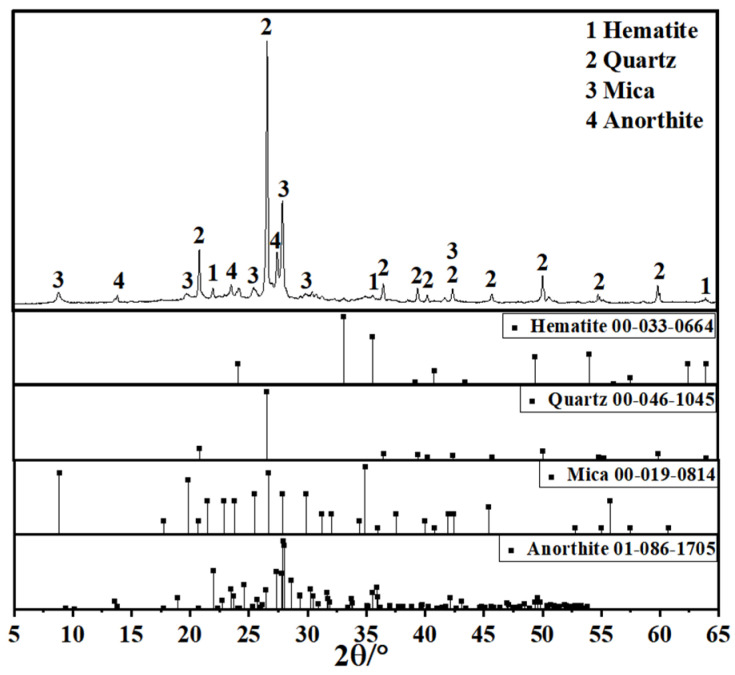
XRD pattern of activated coal gangue.

**Figure 4:**
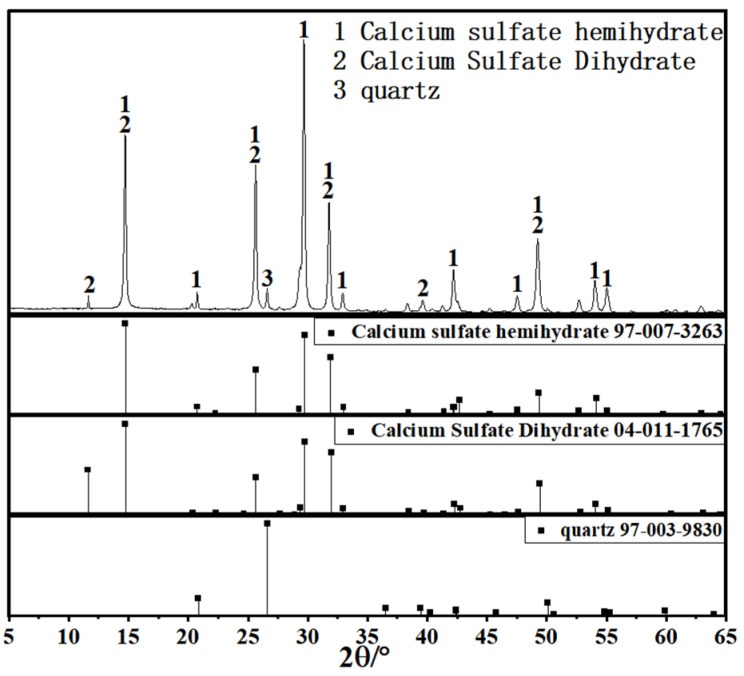
XRD pattern of desulfurized gypsum.

Since coal gangue exhibits relatively low reactivity, a composite activation process was first adopted to prepare activated coal gangue powder (ACG), which served as the main precursor material. Raw coal gangue obtained from the Fuxin coal gangue dump in Liaoning Province was initially crushed into coarse aggregates using a jaw crusher, followed by secondary crushing into fine aggregates with a sand-making machine. The material was then thermally activated in a muffle furnace and subsequently ground into powder with a vertical planetary ball mill for later use. The ACG powder is shown in Figure 1. Commercial S95 slag and fly ash were also selected as supplementary precursor materials. The particle size distributions of the three precursor materials are detailed in Figure 2. The D_50_ values of activated coal gangue, slag, and fly ash are 1.74 μm, 6.11 μm, and 4.17 μm, respectively, while their most probable particle sizes are 1.59 μm, 19.1 μm, and 6.33 μm, respectively. The XRD pattern of ACG (Figure 3) revealed the presence of quartz, anorthite, and mica, with diffraction peaks at 26.6°, 27.4°, and 27.9° corresponding to these phases, respectively. The XRD analysis of desulfurized gypsum (Figure 4) confirmed calcium sulfate (CaSO_4_) as its primary crystalline phase. The activator system used in the experiments comprised desulfurized gypsum (a brownish powder from power plants, purity > 93.0%), sodium hydroxide (white crystals, purity > 96.0%), and waterglass solution (modulus adjusted to 2.0; original modulus 3.3, with Si0, and Na20 contents of 27.3%and 8.54%, respectively, and a Baumé degree of 38.58° Bé). The expansive agents included calcium oxide (CaO, purity > 92.0%, D_50_ = 6.46 μm), magnesium oxide (MgO, purity > 90.0%, D_50_ = 9.26 μm), and calcium sulfoaluminate (C_4_A_3_Š, purity > 90.0%, D_50_ = 2.34 μm). The chemical compositions of the precursors and expansive agents were analyzed via X-ray fluorescence (XRF), with details provided in Table 1. A naphthalene-based high-range water-reducing agent was used as the superplasticizer, with a water-reduction rate of 20% at a content of 0.8%.

#### 2.1.2. Sample Preparation

The symbols C, M, and S represent different types of expansive agents, which refer to calcium oxide (CaO), magnesium oxide (MgO), and calcium sulfoaluminate (C_4_A_3_Š), respectively, as detailed in Table 2. The numbers denote the content; for example, C3 indicates the incorporation of 3% CaO expansion agent. Ordinary Portland cement and CSFG without expansive agents were used as the control groups, while different contents of expansive agents were employed as the modified test groups. The precursor was a ternary blend of activated coal gangue powder, slag, and fly ash in a ratio of 4:5:1. Prior to use, the expansion agent and desulfurized gypsum (with a content of 6%) were dry-mixed with the precursor in a planetary mixer for 3 min. The alkaline activator comprised water glass (2.0 modulus, 9% content) and sodium hydroxide solution (with a content of 5%). A naphthalene-based superplasticizer (0.8% content, 20% water-reduction rate) was also incorporated.

Prism specimens (40 mm × 40 mm × 160 mm) were prepared for compressive and flexural strength testing following the GB/T 17671-2021 Method of Testing Cement Mortar Strength (ISO Method) [16], with a binder-to-sand ratio of 1:3 and a water-to-binder ratio of 0.5. For shrinkage testing, prism specimens (25 mm × 25 mm × 280 mm) were fabricated in accordance with JC/T 603-2004 (Method of Testing Drying Shrinkage of Cement Mortar) [17], using a binder-to-sand ratio of 1:2 and a water-to-binder ratio of 0.45. For microstructural characterization, paste samples were prepared in 40 mm × 40 mm × 40 mm cubic molds and cured under standard conditions (20 ± 2 °C, RH ≥ 95%) until reaching specified ages prior to characterization using XRD, FTIR, SEM-EDS, and BET analyses.

### 2.2. Test Methods

#### 2.2.1. Compressive Strength

Strength tests of prism specimens with dimensions of 40 mm × 40 mm × 160 mm were conducted in accordance with the Chinese National Standard GB/T 17671-2021 Method of Testing Cement Mortar Strength (ISO Method). After curing in a standard curing chamber for 3 d and 28 d, respectively, the specimens were subjected to flexural and compressive strength tests using a WDW-100E (Jinan Times Test Gold, Jinan, China) electro-hydraulic servo universal testing machine. The quantity and dimension of the samples are shown in Table 3.

#### 2.2.2. Drying Shrinkage

Drying shrinkage tests of prism specimens with dimensions of 25 mm × 25 mm × 280 mm were carried out in accordance with the Chinese Building Materials Industry Standard JC/T 603-2004 Test Method for Drying Shrinkage of Cement Mortar. The specimens were first cured and molded in a curing chamber at a temperature of (25 ± 2) °C and relative humidity of (98 ± 2)%. After 24 h, the specimens were demolded and immediately immersed in water for 48 h. Upon removal, the specimen surfaces were carefully wiped with lint-free cloths and the gauge studs were cleaned with alcohol swabs to remove any impurities. A precision electronic balance (capacity 2000 g, accuracy 0.01 g) and a digital length comparator (capacity 30 mm, resolution 0.001 mm) were used to measure the initial mass and baseline length of the specimens, respectively. After the initial measurements, the specimens were transferred to a constant temperature and humidity chamber (temperature 20 ± 3 °C, relative humidity 50 ± 4%) for drying shrinkage curing. The balance and length comparator were then used to measure the mass loss and length change of the specimens at curing ages of 4, 5, 6, 7, 8, 9, 10, 14, 17, 21, 24, 28, 35, 42, 56, 90, 120, 150, and 180 days.

#### 2.2.3. Autogenous Shrinkage

The autogenous shrinkage test method was adapted from the drying shrinkage test procedure (Section 2.2.2). The specimen preparation, curing regime, and testing ages were the same as those in the drying shrinkage test, with the distinction that, after water curing, the specimens were subjected to a 4 h pre-treatment in a constant-temperature drying oven to remove surface moisture. Subsequently, the specimens were hermetically sealed with three layers of polyethylene film (with a thickness of 0.1 mm) to ensure complete isolation from the external environment, and then placed in a constant temperature and humidity chamber.

#### 2.2.4. Setting Time

The initial and final setting times of CSFG paste prepared at standard consistency using a planetary mixer were determined with a Vicat apparatus (accuracy 0.1 mm) in accordance with GB/T 1346-2011 Test Methods for Water Requirement of Normal Consistency, Setting Time and Soundness of the Portland Cement [18].

#### 2.2.5. X-Ray Diffraction Analysis (XRD)

Samples cured to the specified ages were immersed in analytical-grade absolute ethanol for 48 h to terminate hydration, and then dried in a vacuum oven at 40 °C for 24 h until constant weight was achieved Subsequently, the dried samples were ground into powder and sieved through a 75 μm standard square mesh. Phase analysis was conducted using a Shimadzu XRD-6100 (Shimadzu, Shanghai, China) diffractometer with a Cu target. The diffraction angle (2θ) ranged from 5° to 65°, with a scanning rate of 5°/min under continuous scanning mode. Each sample was tested in triplicate to ensure reproducibility, and the phase identification and semi-quantitative analysis were carried out using Jade 9.0 software.

#### 2.2.6. Fourier Transform Infrared Spectroscopy (FTIR)

Molecular structural characterization of the pretreated paste powder samples was performed using a Thermo Scientific Nicolet iS10 FTIR spectrometer (Thermo, Waltham, MA, USA). The test parameters were set to a wavenumber range of 400 to 4000 cm^−1^ with a resolution of 4 cm^−1^.

#### 2.2.7. Scanning Electron Microscopy–Energy Dispersive Spectroscopy (SEM-EDS)

Following the pretreatment procedure detailed in Section 2.2.5, the dried samples were crushed to appropriate sizes and subsequently sputter-coated with a gold layer to prevent surface charge accumulation caused by electron beam bombardment during imaging. Microstructural characterization was then performed using a ZEISS EVO LS15 scanning electron microscope operated at 3 kV, with elemental composition of the reaction products determined by energy-dispersive X-ray spectroscopy (EDS).

#### 2.2.8. Nitrogen Adsorption Method (BET)

Nitrogen adsorption–desorption tests were performed using a 3H-2000PS2 (Quantachrome, Beijing, China) instrument. The vacuum degassing pressure was set at 1 × 10^−3^ mbar, and the relative pressure during the adsorption–desorption process was maintained within the range of 0.001 to 0.998.

#### 2.2.9. Data Analysis and Model Establishment

A geopolymer shrinkage prediction model was developed within the MATLAB (R2024a) environment based on the GL 2000 and EN 1992 frameworks. Subsequently, the model parameters were established by employing nonlinear regression analysis to fit the experimental data. Finally, validation of the model’s accuracy and reliability was performed by comparing its predictions with an independent set of experimental data.

## 3. Discussion of Results

### 3.1. Unconfined Compressive Strength

**Figure 5:**
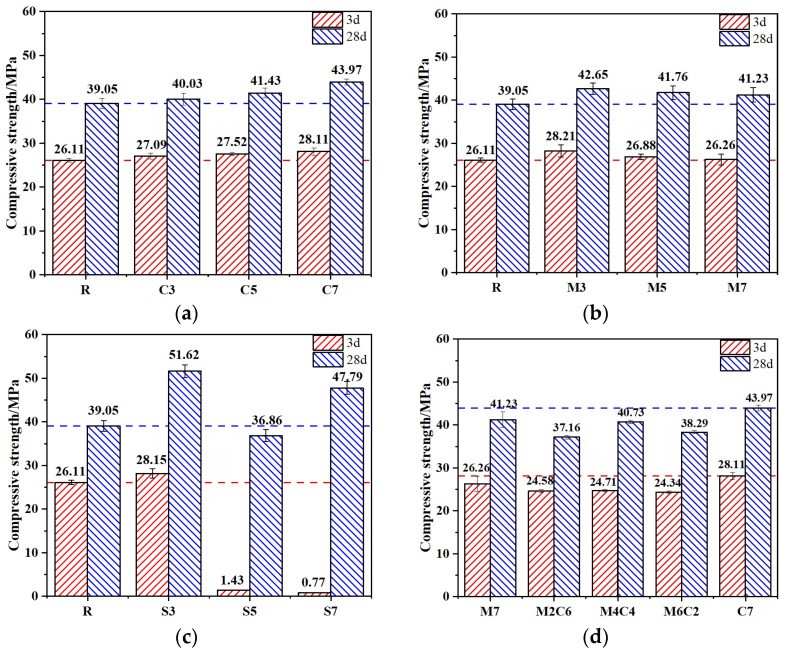
Compressive strength of CSFG mortar specimens: (**a**) CaO, (**b**) MgO, (**c**) C_4_A_3_Š and (**d**) MgO/CaO.

The influence of expansive agent content on the strength development of CSFG mortar is illustrated in Figure 5. As shown in Figure 5a, an upward trend in both 3-day and 28-day compressive strengths was observed with increasing CaO content, although the increments were relatively small. At the 7% CaO content, increases of 7.1% and 12.6% were recorded for 3-day and 28-day compressive strengths, respectively, compared to the reference group, while the maximum 28-day strength of 43.97 MPa was attained. The main reason is that the increase in CaO concentration reduces the relative amount of Na_2_O·nSiO_2_ in the system, thereby increasing the CaO/Na_2_O·nSiO_2_ ratio. This condition promotes the dissolution of coal gangue and fly ash, accelerating the formation of C-A-S-H gels, calcium carbonate, periclase, and other products.

A decreasing trend in both 3-day and 28-day compressive strengths was observed in the MgO system (Figure 5b) as the content was increased from 3% to 7%, although the values remained higher than those of the reference group with only minor reductions. At the 7% MgO content, the compressive strengths were still measured to be 5.7% and 5.6% higher than the reference at 3 days and 28 days, respectively. The late-age strength enhancement was found to be inferior to that of the CaO system, attributed to the presence of an increased MgO crystalline phase. This phase was observed to react with the alkaline activator to form magnesium silicate hydrate (M-S-H), reported to disrupt the stable cross-linked network of C-A-S-H gels [19].

In the C_4_A_3_Š system (Figure 5c), when the C_4_A_3_Š content is 3%, the 3 d and 28 d compressive strengths increase by 7.8% and 32.2%, respectively. However, as the content increases from 3% to 5%, the 3 d compressive strength drops drastically, with reductions of 94.5% and 97%. The primary reason is that increasing C_4_A_3_Š concentration raises the system’s pH, thereby accelerating the hydrolysis reaction of C_4_A_3_Š. This results in the rapid generation of a large amount of expansive product—ettringite (AFt). However, the fast formation of AFt is mismatched with the solidification process of the CSFG gel network. AFt tends to precipitate on the surfaces of precursor particles, partially hindering the progress of geopolymerization and delaying the development of the three-dimensional polymeric network [20]. In addition, at contents of 5% and 7%, C_4_A_3_Š exhibits a rather unique strength development trend. Although the 3 d compressive strengths are only 1.43 MPa and 0.77 MPa, respectively, the 28 d compressive strengths rise rapidly to 36.86 MPa and 47.79 MPa. This indicates that the CSFG reaction continues over time; the later-formed gel phases effectively repair early-stage microstructural defects, while the strength contribution of AFt gradually emerges, together achieving a self-healing effect on mechanical performance. Considering both 3 d and 28 d strength, it is recommended that the C_4_A_3_Š content should not exceed 3%.

As shown in Figure 5d, the effect of MgO content on the compressive strength of CSFG mortar follows a nonlinear trend. The “first increase, then decrease” evolution can be attributed to the shift in the Mg^2+^/Ca^2+^ competitive mechanism. At low MgO concentrations, Ca^2+^ dominates the reactions, promoting the formation of C-A-S-H gels; however, when the MgO concentration becomes excessive, Mg^2+^ participates in the reaction, reducing the formation of strength-enhancing products. The combined action of the two does not produce a synergistic strengthening effect.

### 3.2. Setting Time

**Figure 6:**
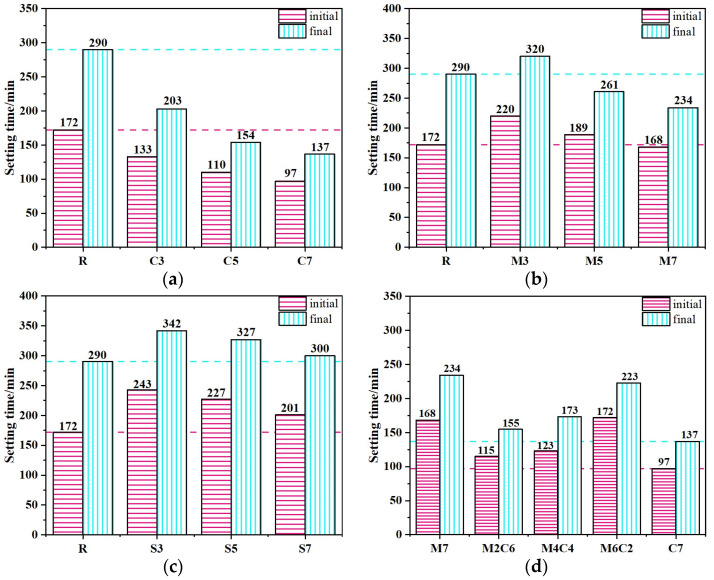
The setting time of CSFG activated by expansive agent: (**a**) CaO, (**b**) MgO, (**c**) C_4_A_3_Š and (**d**) MgO/CaO.

The setting times of CSFG pastes are illustrated in Figure 6. It can be seen from the CaO system (Figure 6a) that both the initial and final setting times of the CaO system pastes decreased significantly with the increase in CaO addition from 3% to 7%, indicating the setting-accelerating characteristic of CaO. When the CaO content was 7%, the initial and final setting times were shortened by 43.6% and 52.8%, respectively. This acceleration effect was attributed to the increased CaO content, which led to greater water consumption used for the formation of Ca(OH)_2_ in the system, reduced the water-cement ratio, and raised the concentrations of Ca^2+^ and [SiO_4_]^4−^ ions, thereby promoting the rapid formation of C-A-S-H gel and enhancing paste coagulation, ultimately shortening the setting time [21]. For the MgO system (Figure 6b), as the MgO content increased from 3% to 7%, the paste setting time showed a downward trend, with a more pronounced reduction in the final setting time. When the MgO content reached 7%, the initial and final setting times were reduced by 2.3% and 19.3%, respectively. This phenomenon was ascribed to MgO’s role as a highly reactive expansive agent with a large specific surface area. Upon increased contact with water, it rapidly underwent a polymerization reaction to form magnesium hydroxide (Mg(OH)_2_). The released heat accelerated the dissolution and polycondensation reaction of activators with raw materials such as activated coal gangue and fly ash in the CSFG, facilitating the formation of an early-strength framework and consequently reducing the setting time [22]. However, in the composite MgO-CaO system (Figure 6d), as the MgO content increased from 2% to 6% and the CaO content decreased from 6% to 2%, both the initial and final setting times exhibited an increasing trend, but they were all shorter than those of the reference group. This behavior primarily resulted from the dual effect of the Mg^2+^/Ca^2+^ ions competition mechanism: at low MgO content, the thermal effect dominated the coagulation-promoting effect, whereas at high MgO content, the ion competition inhibited the coagulation-promoting effect of CaO. In the C_4_A_3_Š system (Figure 6c), as the C_4_A_3_Š content increased from 3% to 7%, both the initial and final setting times of the pastes showed a downward trend, but remained longer than those of the reference group. At a 7% C_4_A_3_Š content, the initial and final setting times were reduced by 16.0% and 3.5%, respectively, and the impact of C_4_A_3_Š on the initial setting time was greater. This was mainly due to the high reactivity of C_4_A_3_Š, where the AFt crystals generated by its polymerization reaction provided heterogeneous nucleation sites for the CSFG gel and further promoted the polymerization of aluminosilicates via a local alkaline microenvironment [23]. Notably, the reference group was observed to exhibit shorter setting time (172 and 290 min), revealing a competition mechanism between expansive stress and gel strength development: during the early polymerization stage, the micro-expansive stress induced by rapid AFt crystal growth exceeded the initial load-bearing capacity of the geopolymer network (3 d compressive strength < 5 MPa), leading to localized structural damage and triggering system reorganization, thereby prolongating the time required to reach a stable solidification state.

In summary, different expansive agents had significant differential effects on the setting time of CSFG: compared with the reference group, the incorporation of MgO and C_4_A_3_Š prolonged the setting time, whereas CaO exhibited a setting-accelerating effect. The differences among the three were primarily attributed to two key mechanisms: firstly, the water consumption effect during the polymerization of expansive agents altered the water-cement ratio of the system, thereby influencing the ion migration rate and the kinetics of early polymerization product formation; secondly, The specific chemical reaction pathways of different expansive agents and the volumetric effects of their products (e.g., AFt, Mg(OH)_2_) exerted specific regulatory effects on the setting process [21,23].

### 3.3. Drying Shrinkage, Autogenous Shrinkage and Mass Loss

#### 3.3.1. Drying Shrinkage

**Figure 7:**
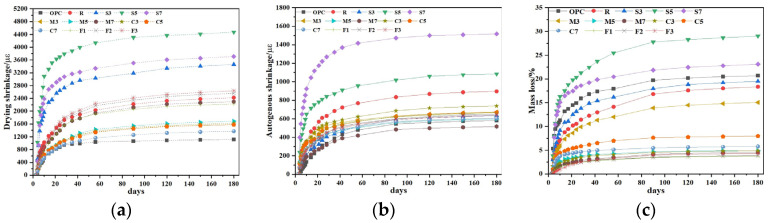
The effect of expansive agent activated CSFG on (**a**) drying shrinkage, (**b**) autogenous shrinkage and (**c**) mass loss.

The influence of different expansive agents on the shrinkage behavior of CSFGs is detailed in Figure 7. Regarding drying shrinkage performance (Figure 7a), the three types of expansive agents, CaO, MgO, and C_4_A_3_Š, were observed to exhibit distinctly different influence patterns with increasing content. At 28 d of age, the CaO group demonstrated more significant shrinkage inhibition. As the CaO content was increased from 3% to 7%, the drying shrinkage was reduced by 36.5%, 38.2%, and 43.5%, respectively, compared to the reference group, with the lowest shrinkage value of 974 με achieved at 7% CaO, which was comparable to cement-based materials of the same age. When the content of 3%, 5%, and 7%, the drying shrinkage was decreased by 38.0%, 37.1%, and 9.4%, respectively. However, within the same range, the content of C_4_A_3_Š increased by 58.3%, 119.7% and 78.5%, respectively. The CaO-MgO composite system exhibited a nonlinear variation: as the proportion of MgO was increased, the drying shrinkage was first reduced by 9.6% and then increased by 4.2%. The above differences essentially reflect the differences in the microscopic mechanisms of action of different polymerization products. The polymerization reaction of CaO was noted to produce a significant volume expansion effect (>250%), and the formed Ca(OH)_2_ crystals effectively counteracted drying shrinkage stress through the action of crystallization pressure [24]. This in situ expansion characteristic established CaO as a key component for improving the volumetric stability of geopolymers. In contrast, the polymerization product of MgO, Mg(OH)_2_, was observed not only to compensate for shrinkage deformation through volume expansion but also to refine the pore structure and reduce material porosity, thereby reducing shrinkage caused by water evaporation [25].

#### 3.3.2. Autogenous Shrinkage

Further analysis of the autogenous shrinkage evolution pattern (Figure 7b) revealed that the development of autogenous shrinkage in geopolymer was significantly correlated with its polymerization process. The three-stage development characteristics (rapid-slow-stable) exactly corresponded to different stages of the CSFG polymerization reaction: the rapid dissolution of aluminosilicates in the initial stage, the gradual strengthening of the gel network in the intermediate stage, and the chemical equilibrium of the system in the later stage [26]. Experimental data indicated that expansive agents exerted a remarkable regulatory effect on the autogenous shrinkage of CSFGs. At 28 d of age, the autogenous shrinkage reduction effects of different expansive agent systems showed obvious differences: when the content of the CaO group was 3%, 5%, and 7%, the reductions were 12.8%, 21.2%, and 29.9%, respectively; for the MgO group at the same contents, the reductions were 16.3%, 30.6%, and 49.5%, respectively; while the C_4_A_3_Š group exhibited a trend of initial decrease followed by an increase. At 180 d of age, the MgO group still maintained the optimal shrinkage inhibition effect, with the lowest shrinkage value of 549 με achieved at 7% MgO content, which was even lower than that of cement-based materials of the same age.

In summary, CaO, MgO, and C_4_A_3_Š expansive agents were demonstrated to inhibit the shrinkage of CSFGs, and their inhibitory effects are closely related to the content and type. The inhibitory effect mainly stems from the dual effects of the polymerization products of the expansive agent: on the one hand, it compensates for the shrinkage stress through volumetric expansion; on the other hand, the internal pore structure is filled through crystal growth. It was notably observed that when the C_4_A_3_Š content exceeded 3%, the rapid formation of AFt led to localized stress concentration and microcracking, which consequently diminished its compensatory effect on autogenous shrinkage [19]. Compared to alkali metal activators, alkaline earth metal activators (CaO, MgO, and C_4_A_3_Š) were found to exhibit lower pH values, which endowed them with a higher safety factor in engineering construction. Additionally, by reducing the dependency of precursor materials on alkali metals, the risk of geopolymer weathering was effectively controlled, thereby significantly enhancing the long-term durability of the material.

#### 3.3.3. Mass Loss

The influence of laws of different expansive agents on the mass of CSFG mortar under a 50% relative humidity (RH) environment is detailed in Figure 7c. Experimental data indicated that mass loss was observed to increase nonlinearly with curing time, with over 50% of the total loss occurring within the first 28 d. Comparison among different expansive agent systems revealed that samples with single incorporation of MgO and CaO exhibited significantly lower cumulative mass loss at 180 d than the C_4_A_3_Š system, whereas the composite system demonstrated higher mass loss. Notably, despite similar mass loss values at 180 d for 3% MgO and 7% CaO contents, a significant difference of 10% was observed in the 28 d drying shrinkage values. This non-corresponding relationship indicated that mass loss was not the sole factor determining drying shrinkage, which aligned with the multi-factor influence mechanism proposed by Collins [27]. That is, the pore size distribution characteristics, crystal properties, and gel characteristics regulate the shrinkage behavior of the material.

### 3.4. Polymerization Products and Microstructure

#### 3.4.1. XRD

**Figure 8:**
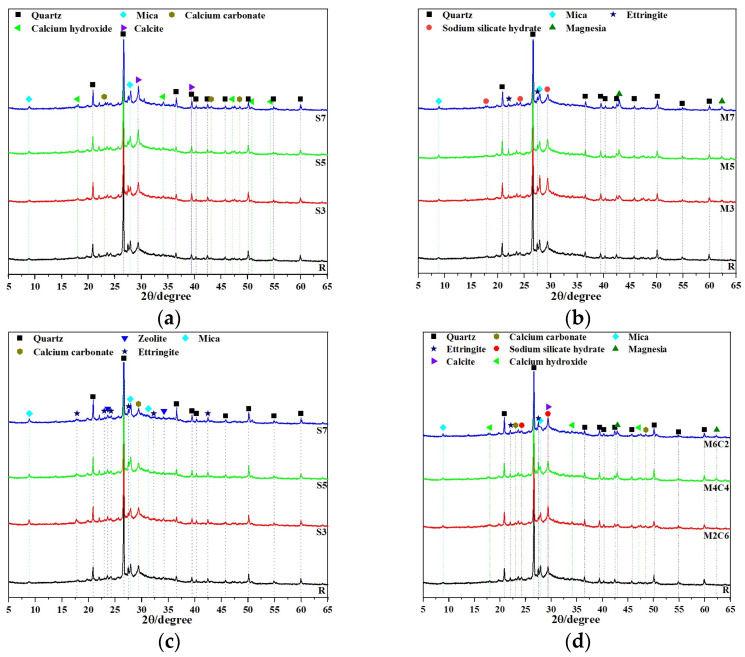
XRD of CSFG activated by expansive agent: (**a**) CaO, (**b**) MgO, (**c**) C_4_A_3_Š and (**d**) MgO/CaO.

XRD phase analysis revealed the influence of different expansive agents on the crystallization behavior of the CSFG system, as shown in Figure 8. The results indicated that the quartz phase (PDF: 00-046-1045) was detected in all CSFG samples, with its diffraction peaks located at 20.86°, 26.64°, 36.54°, and 50.14°. This phase is derived from the inactive components in the activated coal gangue raw materials [28]. The CSFG without expansive agents is mainly amorphous C-S-H, C-A-S-H gels and AFt as the main polymerization products. Due to the highly amorphous nature of C-S-H and C-A-S-H gel, a weak background peak appears in the 2θ range from 25° to 35° in Figure 8 [29]. Due to the change in the type of expansive agents, the detected crystal phase composition also shows significant differences.

In-depth analysis of the CaO system (Figure 8a) revealed that the incorporation of CaO significantly promoted the carbonation reaction of the CSFG, with C5 displaying the highest intensity calcite diffraction peaks. Meanwhile, the characteristic peaks of calcium carbonate located at 23.05° and 48.50° further confirmed that Ca(OH)_2_ followed a dual consumption pathway during the setting process: one port participated in the polymerization reaction to form C-S-H gel, and the other part underwent carbonation with atmospheric CO_2_ [30]. The aforementioned competition mechanism ultimately determined the phase composition of the CSFG system.

Significantly different characteristics are exhibited in the phase evolution of the MgO system, as shown in Figure 8b. With the increase in MgO content, the diffraction peak intensities of the mica phase (2θ = 27.86°) and sodium silicate hydrate (2θ = 29.36°) were significantly reduced and accompanied by a slight right-shift, while the characteristic peak intensity of the periclase phase (2θ = 42.94°) was correspondingly enhanced. On one hand, the Mg^2+^ released by dissolution of the active component altered the solution chemistry by consuming OH^−^ and H_2_O, thereby inhibiting the dissolution and reprecipitation processes of Al^3+^ and Si^4+^; on the other hand, it acted as a heterogeneous nucleation site promoting the formation of an amorphous gel phase. This synergistic effect resulted in a reduction in the content of crystalline mica and sodium silicate hydrate, and collectively regulated the microstructure development by optimization of the gel network structure and refinement of the pore distribution (BET tests indicated a 10% reduction in the average pore size) [12].

Significantly different phase evolution characteristics were observed in the C_4_A_3_Š system (Figure 8c), where the characteristic calcium carbonate peak intensity at 2θ = 29.41° showed an upward trend with increasing content, while the diffraction intensity of the AFt phase at 2θ = 27.54° displayed a nonlinear pattern characterized by an initial decrease followed by an increase. The phase evolution law of this material originates from the following competitive path: (1) At the low content stage (3%), the coordination competition between SO_4_^2−^ and Ca^2+^ from desulfurized gypsum resulted in hindered heterogeneous nucleation of AFt; (2) After the content exceeded the critical threshold (5%), the Al^3+^ released by C_4_A_3_Š hydrolysis and SO_4_^2−^ formed a thermodynamically stable AFt phase according to the stoichiometric ratio (3:1), while its decomposition reaction was suppressed [19]. The continuous consumption of Ca(OH)_2_ by C_4_A_3_Š caused a decrease in the system pH, which consequently accelerated the carbonation process and enhanced the crystallinity of calcium carbonate.

For the MgO-CaO composite system (Figure 8d), the phase evolution was observed to exhibit a significant synergistic effect: with increasing MgO addition, the diffraction peak intensity of the mica phase was significantly reduced, while the characteristic peaks of AFt and MgO demonstrated a trend of initial increase followed by decrease, whereas the composite peak of calcite-sodium silicate hydrate at 29.42° remained relatively stable. At low MgO content, the reaction was dominated by Ca^2+^, promoting the formation of AFt and MgO crystalline phases; at high content, the participation of Mg^2+^ in the reaction inhibited the generation of the aforementioned crystalline phases, revealing the complex crystallization behavior of multi-ion synergy in the composite expansion system.

#### 3.4.2. FTIR

**Figure 9:**
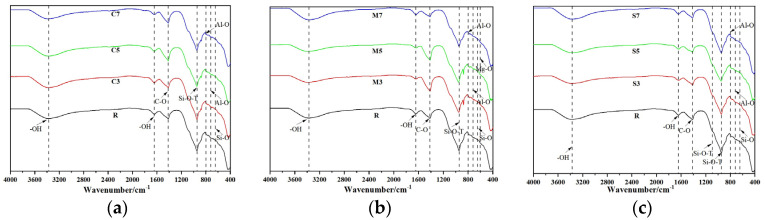
FTIR of expansive agent activated CSFG: (**a**) CaO, (**b**) MgO and (**c**) C_4_A_3_Š.

FTIR spectroscopic analysis was conducted to reveal the key structural characteristics of the CSFG, as detailed in Figure 9. The characteristic peaks observed in the ranges of 3366–3389 cm^−1^ and 1641–1648 cm^−1^ were attributed to the asymmetric stretching vibration of -OH groups [31]. The C-O asymmetric stretching vibration peak detected at 1413–1418 cm^−1^ indicated that carbonation reactions of varying degrees occur [32]. The broadband peak at 1082–1097 cm^−1^ can be attributed to the asymmetric stretching vibration of the Si-O-T (T = Si/Al) tetrahedral structure [33]. The spectral bands in the 712–717 cm^−1^ and 797–801 cm^−1^ intervals correspond, respectively, to the stretching vibrations of Al-O [34]. The characteristic peak found at 638–651 cm^−1^ reflected the deformation vibration of Si-O in the C-A-S-H gel [35]. The characteristic peak near 604 cm^−1^ indicates the bending vibration of the Mg-O bond [36].

Further comparison of the spectra from different groups revealed that in the C3-C7 groups, the main Si-O-T peak was observed to shift from 944 cm^−1^ to 949 cm^−1^, confirming that enhanced slag dissolution promoted an increase in the Ca/Si ratio of the C-A-S-H gel (EDS showed that it rose from 1.44 to 1.80). In the M3-M7 groups, the Mg-O vibration peak migrated from 604 cm^−1^ to 600 cm^−1^, reflecting that the enhanced dissolution of Mg^2+^ inhibited the dissolution of [AlO_4_]^5−^ and [SiO_4_]^4−^. In the S3-S7 groups, the characteristic peak of calcium carbonate was noted to shift toward lower frequency from 1416 cm^−1^ to 1413 cm^−1^, indicating an increase in its polymerization degree, a change closely related to the later strength development of the material; The vibration peak of Al-O in AFt shifted from 714 cm^−1^ to 717 cm^−1^, confirming that enhanced dissolution of Al^3+^ and SO_4_^2−^ promoted the crystallization of AFt.

#### 3.4.3. SEM-EDS

**Figure 10:**
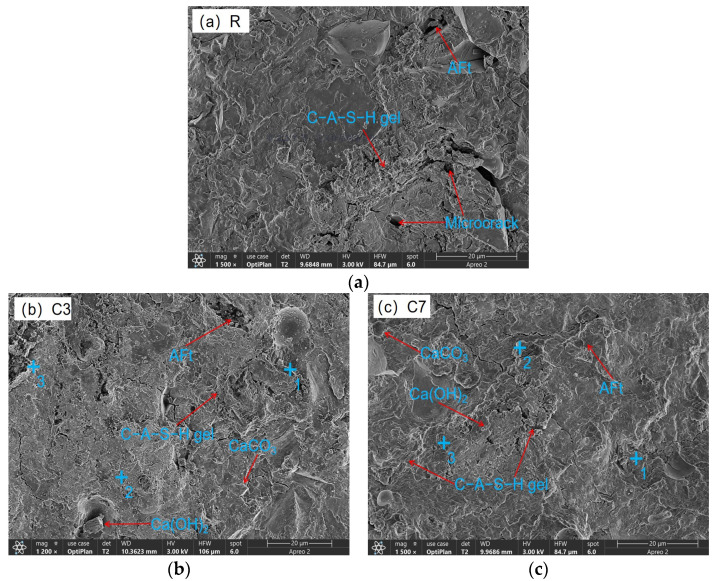

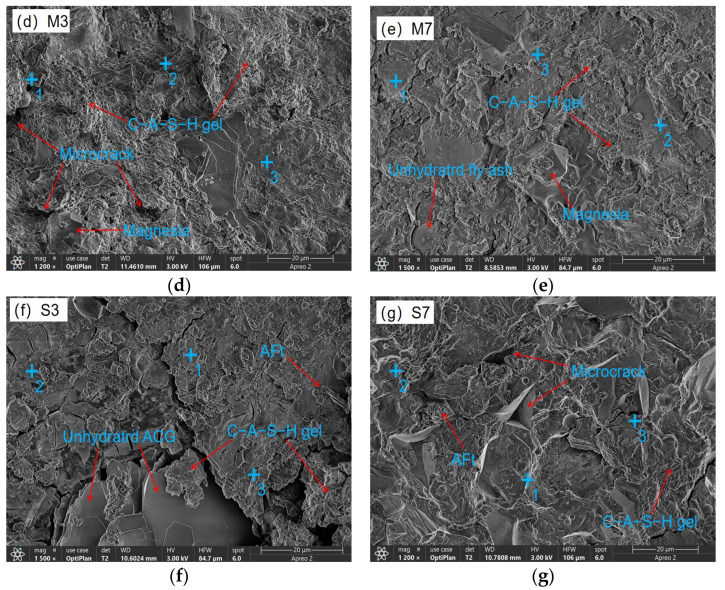
SEM-EDS of expansive agent activated CSFG: (**a**) R, (**b**) C3, (**c**) C7, (**d**) M3, (**e**) M7, (**f**) S3 and (**g**) S7.

The microstructural characteristics of 28 d CSFG specimens are shown in Figure 10. SEM analysis indicated that all samples contained incompletely hydrated slag, fly ash, and activated coal gangue particles, whose surfaces were coated with C-A-S-H gel. Compared with the control group, more abundant polymerization products were observed in the experimental group samples. This was primarily attributed to the diversification of reaction pathways caused by the introduction of expansive agents, thereby altering the polymerization process. Obvious microcracks and dispersed hydration products were observed in sample S3, significantly increasing the number of pores. Such microcracks have also been reported in other samples, and their formation is mostly related to moisture loss during drying. Notably, the C-A-S-H gel network was found to fill pores while simultaneously covering the surfaces of unhydrated particles, effectively increasing the interfacial contact area. In contrast, C7 presents a more interlaced and stacked disordered C-A-S-H gel and Ca(OH)_2_ crystal. This microstructure feature is closely related to the formation of secondary compressive strength, which shows in macroscopic mechanical behavior. The aforementioned observations were consistent with the mechanical property test data.

The coexistence of C-A-S-H and C-N-A-S-H gels was confirmed in all CSFG samples by the detection of characteristic elements such as Na, Si, Al, and Ca in the EDS analysis results (Table 4) [37]. In samples C3 and C7, hexagonal lamellar Ca(OH)_2_ crystals were observed to form a tightly interwoven microstructure with C-A-S-H gel. This crystalline phase effectively dissipated the shrinkage stress of the C-A-S-H gel through a stress buffering mechanism resembling micro-agglomeration [1,11], thereby significantly reducing the drying shrinkage of the CaO system (the decline reached 43.49%). This effect was primarily attributed to its crystalline expansion characteristics and interfacial synergy with the gel phase. The MgO system exhibited a distinct ion substitution effect: the Ca/Si ratio in the M7 group was reduced to 0.86 (compared to 1.06 in the M3 group), while the Mg/Si ratio increased to 0.17, indicating that Mg^2+^ competition inhibited C-A-S-H precipitation and confirming the synergistic coexistence of C-A-S-H and M-S-H gel phases. For the C_4_A_3_Š system, the symbiotic structure of rod-like AFt crystals and C-A-S-H gel could effectively inhibit shrinkage; however, excessive addition will lead to (1) weakened interfacial transition zones due to accelerated polymerization and (2) the drying shrinkage and autogenous shrinkage increased significantly (by 78.92% and 100.90%, respectively).

### 3.5. BET

**Figure 11:**
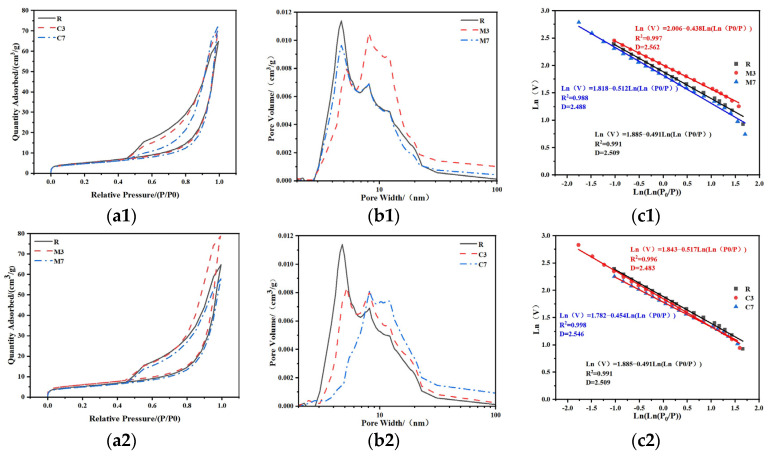
BET pore distribution analysis: (**a**) absorption and desorption curves, (**b**) pore volume diagram, (**c**) fractal dimension.

The BET test results are detailed in Figure 11 and Table 5. All 28 d cured CSFGs exhibited typical Type IV isotherms and Type H3 hysteresis loops (Figure 11a), confirming that their pore structures were dominated by mesopores. According to the IUPAC classification standard [38], the pore size distribution can be divided into micropores (<2 nm), mesopores (2–50 nm), and macropores (>50 nm). Due to the instrument’s pressure upper limit (P/P_0_ = 0.995), mainly micropores and mesopores were detected in this study. Quantitative analysis (Figure 11b) indicated that the type and content (3–7%) of expansive agents synergistically regulated the evolution of the pore structure: high concentrations of CaO increased the average pore size from 2.43 nm to 2.62 nm and raised the total pore volume from 1.08 cm^3^/g to 1.12 cm^3^/g; whereas MgO exhibited the opposite trend, reducing the average pore size from 2.25 nm to 2.04 nm and decreasing the total pore volume to 0.90 cm^3^/g. The pore size distribution curves further revealed that the R and M7 groups showed a single peak at 5 nm, while the M3, C3, and C7 groups exhibited a main peak at 8 nm. In particular, the C7 sample formed a broad bimodal distribution in the 7–12 nm range, a phenomenon attributed to a significant proliferation of pores within this interval, ultimately leading to an increase in its average pore volume.

The fractal dimension (D) was quantitatively analyzed using the Frenkel–Halsey–Hill (FHH) model [39] to characterize the surface complexity of the porous materials. Regression results indicated that the h values of all samples fell outside the range of −1/3 to 0 (Figure 11c), confirming that surface tension was the dominant force in the nitrogen adsorption process. The test results show that a higher fractal dimension corresponds to more significant pore structure heterogeneity and a wider pore size distribution. This result provides a new theoretical perspective for understanding the structure-property relationship of geopolymer.

Incorporating expansive agents has a significant regulatory effect on D of CSFGs. The fractal dimension of the 7% MgO sample was the lowest (D = 2.488), followed by the control group (D = 2.509), while the 3% MgO sample reached the peak (D = 2.562). At high MgO content (7%), the alkali-activated reaction was promoted to form magnesium silicate hydrate (M-S-H), selectively filled mesopores larger than 10 nm, leading to pore geometry homogenization and reduced topological complexity. The simultaneously formed dense C-A-S-H gel (Ca/Si = 0.86) further weakens the pore structure heterogeneity. In contrast, low MgO content (3%) resulted in partial replacement of Ca^2+^ by Mg^2+^ in C-A-S-H (Mg/Si = 0.21), causing gel network distortion and micropore proliferation. Coupled with the incomplete phase formation of M-S-H gel, local MG-rich regions are formed, ultimately increasing the disorder of the hierarchical pore structure. However, the CaO sample exhibited an opposite trend to that of MgO: the incorporation of CaO induced more rapid and pronounced coarsening of the pore structure, resulting in an increased fractal dimension. This effectively reduced the capillary pressure, thereby inhibiting drying shrinkage through this mechanism.

### 3.6. Shrinkage Prediction Model

#### 3.6.1. Drying Shrinkage Prediction Model

Currently widely used drying shrinkage prediction models, such as ACI 209 [40], CEB-FIP 1990 [41], and GL 2000 [42], etc., are primarily established based on environmental parameters and specimen geometric characteristics, and have demonstrated good applicability in traditional Portland cement systems. However, these models have significant biases in predicting the contraction behavior of geopolymers, mainly manifested as: (1) the early-age shrinkage rate was severely underestimated, and (2) the high total shrinkage characteristics of geopolymers could not be accurately reflected. This limitation stems from the fact that the existing models have not fully considered the unique rapid hydration kinetics and microstructure evolution mechanism of geopolymers. Therefore, this study was designed to establish an improved drying shrinkage prediction model suitable for CSFG systems.

Based on the framework of the GL 2000 model, an improved drying shrinkage prediction model suitable for CSFG systems was constructed by introducing the contents of CaO, MgO, and C_4_A_3_Š expansive agents as key variables. The drying shrinkage prediction model for CSFGs is established using MATLAB software. The drying shrinkage expressions are shown as follows ((1) to (7)):

(1) $$\varepsilon_{d}\left(t\right)=\varepsilon_{du}\cdot \beta \left(RH\right)\cdot \beta_{d}\left(t\right)$$

(2) $$\varepsilon_{du}=1437\cdot {\left(\frac{100}{f_{cu}}\right)}^{0.5}\cdot k_{1}\cdot k_{2}\cdot k_{3}\cdot 10^{-6}$$

(3) $$k_{1}=1-0.1380\alpha +0.0119\alpha^{2}$$

(4) $$k_{2}=1-0.1889\beta +0.0258\beta^{2}$$

(5) $$k_{3}=1+0.3579\gamma -0.0334\gamma^{2}$$

(6) $$\beta \left(RH\right)=1+1.18{\left(0.01RH\right)}^{4}$$

(7) $$\beta_{d}\left(t\right)={\left(\frac{t-t_{0}}{t-t_{0}+0.44{\left(V/S\right)}^{2}}\right)}^{0.659}$$

where ε_du_ represents the final drying shrinkage value of the specimen, με; β(RH) represents the relative humidity development function; β_d_(t) represents the function of drying shrinkage with respect to age; t days; t_0_ represents the time when shrinkage testing commenced, d; f_cu_ represents the cube compressive strength of the CSFG at 28 d of age, MPa; V/S represents the surface-to-volume ratio of the CSFG; RH represents the ambient relative humidity, %; k_1_ represents the influence coefficient of CaO expansive agent; k_2_ represents the influence coefficient of MgO expansive agent; k_3_ represents the influence coefficient of C_4_A_3_Š expansive agent; α represents the content of CaO expansive agent, %; β represents the content of MgO expansive agent, %; γ represents the content of C_4_A_3_Š expansive agent, %.

**Figure 12:**
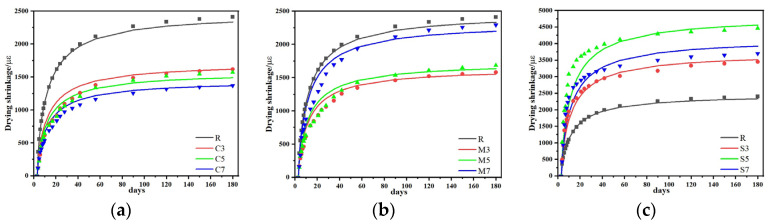
Comparison of predicted and experimental values of drying shrinkage model: (**a**) CaO, (**b**) MgO and (**c**) C_4_A_3_Š.

Good agreement was demonstrated between the predicted values from the CSFG drying shrinkage model developed in this study and the experimentally measured results, as shown in Figure 12. Correlation analysis (Table 6) indicated that the correlation coefficients between the model predictions and measured data all exceeded 0.90, validating that the model can accurately characterize the time-dependent characteristics of drying shrinkage in CSFGs, while also confirming the rationality of the model parameter selection and the reliability of the prediction results.

#### 3.6.2. Autogenous Shrinkage Prediction Model

Currently existing autogenous shrinkage prediction models, including the Japanese standard [43], CEB-FIP 1990 [41], and EN 1992 [44], etc., exhibit significant inadequacies in their applicability to geopolymer material systems. These are primarily manifested as: (1) the autogenous shrinkage values were severely underestimated, and (2) the rapidly developing shrinkage characteristics could not be accurately represented. Although the absolute value of autogenous shrinkage in geopolymers constitutes only 20–50% of the drying shrinkage, it still exerts an important influence on the long-term performance of the material. Therefore, this study was designed to establish an improved autogenous shrinkage prediction model suitable for CSFG systems.

Based on the framework of the EN 1992 model, an improved autogenous shrinkage prediction model suitable for CSFG materials was constructed by introducing the contents of CaO, MgO, and C_4_A_3_Š expansive agents as key variables. Using the experimental data from low-shrinkage geopolymer systems, multiple nonlinear regression analysis was performed with MATLAB, ultimately establishing the autogenous shrinkage prediction Equations (8)–(13):

(8) $$\varepsilon_{a}\left(t\right)=\varepsilon_{au}\cdot \beta_{a}\left(t\right)$$

(9) $$\varepsilon_{au}=31.45\cdot \left(f_{cu}-10\right)\cdot k_{1}\cdot k_{2}\cdot k_{3}\cdot 10^{-6}$$

(10) $$k_{1}=1-0.0352\alpha -0.003\alpha^{2}$$

(11) $$k_{2}=1-0.1008\beta +0.0045\beta^{2}$$

(12) $$k_{3}=1-1.1046\gamma +0.4136\gamma^{2}-0.0352\gamma^{3}$$

(13) $$\beta_{a}\left(t\right)=1-\exp\left(-0.178{\left(t-t_{0}\right)}^{0.583}\right)$$

where εa(t) represents the shrinkage value of the specimen at an age of t days, με; εau represents the final shrinkage value of the specimen, με; βa(t)) represents the function describing the development of autogenous shrinkage with age; fcu represents the cube compressive strength of the CSFG at 28 d of age, MPa; k_1_ represents the influence coefficient of CaO expansive agent; k_2_ represents the influence coefficient of MgO expansive agent; k_3_ represents the influence coefficient of C_4_A_3_Š expansive agent; α represents the content of CaO expansive agent, %; β represents the content of MgO expansive agent, %; γ represents the content of C_4_A_3_Š expansive agent, %.

**Figure 13:**
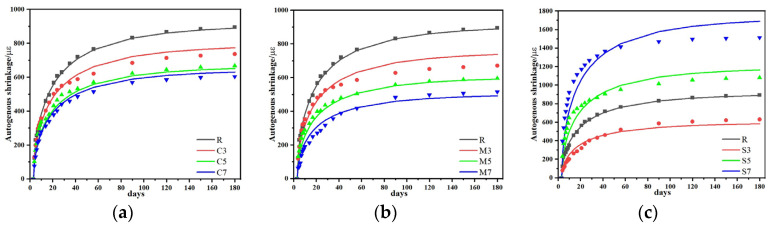
Comparison of predicted and experimental values of autogenous shrinkage model: (**a**) CaO, (**b**) MgO and (**c**) C_4_A_3_Š.

Good agreement was demonstrated between the predicted values of the autogenous shrinkage model developed in this study and the experimentally measured data, as shown in Figure 13. According to the correlation analysis results (Table 7), the correlation coefficients between the model predictions and measured values all exceeded 0.90, indicating that the model can effectively characterize the development pattern of autogenous shrinkage in CSFGs, while also validating the rationality of the model parameter settings and the accuracy of the prediction results.

#### 3.6.3. Shrinkage Model Validation

To further verify the reliability and general applicability of the proposed prediction models, the optimal mix proportion group was re-tested based on the modified GL 2000 and EN 1992 models, and supplemented with CSFG shrinkage data from Tao, Zhang et al. [12,13,45,46,47]. Multiple nonlinear regression analyses were then conducted using MATLAB.

**Figure 14:**
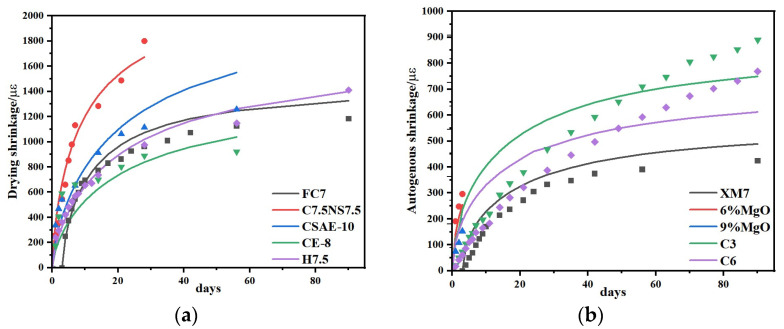
Comparison of predicted and experimental values of shrinkage model: (**a**) drying shrinkage and (**b**) autogenous shrinkage [12,13,45,46,47].

Strong agreement was demonstrated between the shrinkage prediction model developed in this study and the experimental data, as shown in Figure 14. As presented in Table 8, the correlation coefficients between the model predictions and the measured values of the re-tested optimal mix proportion group exceeded 0.9, while those corresponding to data from the literature were above 0.75. These results demonstrate the model’s capability to reliably capture the shrinkage evolution of CSFG materials, thereby validating both the parameterization and predictive accuracy of the proposed approach.

## 4. Conclusions

(1) The type and content of expansive agent had a certain effect on the compressive strength of CSFG. With single incorporation of CaO, the compressive strength exhibited an increasing trend as the content rose. When the content of CaO was 7%, 3 d and 28 d compressive strengths were, respectively, increased by 7.1% and 12.6% compared to the reference group. In the meanwhile, 28 d compressive strength was 43.97 MPa, meeting the strength grade requirement of 42.5# cement. With single incorporation of MgO, 3 d and 28 d compressive strengths showed a decreasing trend as the content increased, but the reductions were minor, and both remained higher than those of the reference groups. The lowest compressive strength was observed at 7% content, yet 3 d and 28 d strengths still increased by 5.7% and 5.6%, respectively, relative to the reference group. With single incorporation of C4A3Š, at 3% content, 3 d and 28 d compressive strengths increased by 7.8% and 32.2%, respectively. However, when the content exceeded 3%, 3 d compressive strength decreased sharply; at 5% content, the compressive strength was only 1.43 MPa, a reduction of 94.5%, and at 7% content, it was merely 0.77 MPa, a reduction of 97%. The 28 d compressive strength development rate was relatively high, and the 28 d compressive strength was essentially equivalent to that of the reference group.
(2) The type and content of expansive agents significantly affected the setting time of CSFG paste. With single incorporation of CaO, both the initial and final setting times decreased as the CaO content increased; at 7% CaO content, the initial and final setting times were, respectively, shortened by 43.6% and 52.8%. With single incorporation of MgO, the setting time decreased with increased MgO content, with a more pronounced reduction in the final setting time. At 7% MgO content, the initial and final setting times were reduced by 2.3% and 19.3%, respectively, compared to the reference group. With single incorporation of C4A3Š, the setting time decreased with increasing content, but remained longer than that of the reference group. At 7% content, the initial and final setting times were reduced by 16.0% and 3.5%, respectively, relative to the reference.
(3) The type and content of expansive agents significantly influenced the shrinkage of CSFGs. Single incorporation of CaO demonstrated notable shrinkage inhibition effects. At CaO contents of 3%, 5%, and 7%, the drying shrinkage was, respectively, reduced by 36.5%, 38.2%, and 43.5%, and the autogenous shrinkage was, respectively, reduced by 12.8%, 21.2%, and 29.9%, compared to the reference group. At 7% CaO content, the drying shrinkage value was 974 με, which was comparable to that of cement-based materials of the same age. For MgO contents of 3%, 5%, and 7%, the drying shrinkage was reduced by 38.0%, 37.1%, and 9.4%, respectively, and the autogenous shrinkage was reduced by 12.8%, 21.2%, and 29.9%, respectively. At 7% MgO content, the drying shrinkage value was 549 με, indicating excellent shrinkage inhibition. For C4A3Š contents of 3%, 5%, and 7%, the shrinkage first decreased and then increased; compared to the reference group, the drying shrinkage increased by 58.3%, 119.7%, and 78.5%, respectively, and the autogenous shrinkage increased by −35.7%, 33.1%, and 100.9%, respectively.
(4) Microscopic analysis indicated that the hydration products of different expansive agent systems varied significantly. C4A3Š primarily promoted the formation of AFt, MgO induced the formation of the M-S-H phase, and CaO mainly produced Ca(OH)_2_ crystals. The shrinkage inhibition effect was primarily determined by the dual effects of the polymerization products of expansive agents: firstly, volumetric expansion compensated for shrinkage stress, and secondly, crystal growth filled the internal pore structure.
(5) Using MATLAB software, the GL-2000 and EN-1992 models were employed, and expansive agent type-specific coefficients were introduced to perform multiple nonlinear regression fitting on the CSFG shrinkage test data. An improved shrinkage prediction model for CSFGs was developed, and the predicted results demonstrated good agreement with the measured results, indicating that the established CSFG shrinkage model was effective and feasible.

## Figures and Tables

**Table 1:** The main chemical composition of precursor and expansion agent (w%).

Composition	SiO_2_	Al_2_O_3_	Fe_2_O_3_	MgO	CaO	K_2_O	Na_2_O	SO_3_
Activated coal gangue	64.73	18.63	5.00	1.98	2.08	3.52	1.47	1.23
Slag	33.65	16.77	1.12	6.51	37.65	0.56	0.71	1.44
Fly ash	62.02	11.29	5.19	1.56	6.07	1.63	0.13	1.04
CaO	2.53	1.54	1.39	0.85	92.11	0.42	0.12	0.05
MgO	4.22	1.56	0.47	90.16	2.85	0.11	0.09	0.33
C_4_A_3_Š	5.63	12.65	1.36	0.85	49.84	0.48	0.05	28.97

**Table 2:** Mix proportion of ASFG slurry.

	Mixture	Activated Coal Gangue Content (%)	Slag Content (%)	Fly Ash Content (%)	CaO Content (%)	MgO Content (%)	C_4_A_3_Š Content (%)
Name							
R							
C3, C5, C7		40	50	10	3, 5, 7		
M3, M5, M7						3, 5, 7	
S3, S5, S7							3, 5, 7
M2C6, M4C4, M6C2					6, 4, 2	2, 4, 6	

**Table 3:** Sample quantity and dimension.

Experimental Name	Sample Quantity	Sample Dimension (mm)
Compressive strength	13 × 6	40 × 40 × 160
Drying shrinkage	14 × 3	25 × 25 × 280
Autogenous shrinkage	14 × 3	25 × 25 × 280
XRD, FTIR, SEM-EDS, BET	13 × 1	40 × 40 × 40

**Table 4:** EDS element table of expansive agent activated CSFG (w%).

Element	C3	C7	M3	M7	S3	S7
O	44.7	48.2	48.0	44.3	47.5	47.1
Ca	23.2	17.3	12.8	13.3	14.3	13.8
Si	9.3	9.6	12.1	15.4	13.3	12.5
C	10.5	12.2	12.4	9.5	8.9	9.8
Al	3.9	4.4	5.4	5.6	5.3	6.9
Na	3.7	4.3	3.8	4.4	5.1	3.9
Mg	1.1	1.4	2.5	2.6	2.0	2.0
Fe	0.9	0.7	1.0	1.8	1.3	1.4
S	1.9	1.4	1.0	1.7	1.1	1.2

**Table 5:** BET data sheet of geopolymer activated by expansive agent.

Name	Pore Volume (cm^3^/g)	Average Pore Diameter (nm)	Surface Area (cm^3^/g)
R	0.085	2.14	19.563
M3	0.108	2.25	21.930
M7	0.076	2.04	17.821
C3	0.090	2.43	18.734
C7	0.094	2.62	16.398

**Table 6:** Correlation coefficient of drying shrinkage contrast curve.

Name	R	C3	C5	C7	M3	M5	M7	S3	S5	S7
R^2^	0.997	0.934	0.969	0.962	0.968	0.954	0.956	0.975	0.902	0.936

**Table 7:** Correlation coefficient of Autogenous shrinkage contrast curve.

Name	R	C3	C5	C7	M3	M5	M7	S3	S5	S7
R^2^	0.996	0.973	0.977	0.989	0.963	0.980	0.965	0.965	0.931	0.905

**Table 8:** The correlation coefficient of contraction contrast curve.

Name	C7.5NS7.5	CSAE-10	6%MgO	9%MgO	CE-8	H7.5	C3	C6	FC7	FM7
R^2^	0.965	0.805	0.768	0.754	0.863	0.983	0.815	0.809	0.937	0.910

## Data Availability

The original contributions presented in this study are included in the article. Further inquiries can be directed to the corresponding author.
